# Estimation of the prevalence and incidence of motor neuron diseases in two Spanish regions: Catalonia and Valencia

**DOI:** 10.1038/s41598-021-85395-z

**Published:** 2021-03-18

**Authors:** Maria A. Barceló, Mònica Povedano, Juan F. Vázquez-Costa, Álvaro Franquet, Marta Solans, Marc Saez

**Affiliations:** 1 Research Group On Statistics, Econometrics and Health (GRECS), University of Girona, Carrer de la Universitat de Girona 10, Campus de Montilivi, 17003 Girona, Spain; 2 CIBER of Epidemiology and Public Health (CIBERESP), Madrid, Spain; 3 Unidad Funcional de Motoneurona (UFMN), Instituto de Investigación Biomédica de Bellvitge (IDIBELL), Hospital Universitario de Bellvitge, L’Hospitalet de Llobregat, Barcelona, Spain; 4 Unidad Funcional de Motoneurona (UFMN), Instituto de Investigación Sanitaria La Fe (IIS La Fe), Hospital Universitario y Politécnico La Fe, Valencia, Spain; 5 Centro de Investigación Biomédica en Red de Enfermedades Raras (CIBERER), Madrid, Spain; 6 Medicine Department, Facultad de Medicina, University of Valencia, Valencia, Spain; 7 Fundació Salut Empordà, Figueres, Spain

**Keywords:** Neurology, Risk factors

## Abstract

According to the degree of upper and lower motor neuron degeneration, motor neuron diseases (MND) can be categorized into amyotrophic lateral sclerosis (ALS), primary lateral sclerosis (PLS) or progressive muscular atrophy (PMA). Although several studies have addressed the prevalence and incidence of ALS, there is a high heterogeneity in their results. Besides this, neither concept has been previously studied in PLS or PMA. Thus, the objective of this study was to estimate the prevalence and incidence of MND, (distinguishing ALS, PLS and PMA), in the Spanish regions of Catalonia and Valencia in the period 2011–2019. Two population-based Spanish cohorts were used, one from Catalonia and the other from Valencia. Given that the samples that comprised both cohorts were not random, i.e., leading to a selection bias, we used a two-part model in which both the individual and contextual observed and unobserved confounding variables are controlled for, along with the spatial and temporal dependence. The prevalence of MND was estimated to be between 3.990 and 6.334 per 100,000 inhabitants (ALS between 3.248 and 5.120; PMA between 0.065 and 0.634; and PLS between 0.046 and 1.896), and the incidence between 1.682 and 2.165 per 100,000 person-years for MND (ALS between 1.351 and 1.754; PMA between 0.225 and 0.628; and PLS between 0.409–0.544). Results were similar in the two regions and did not differ from those previously reported for ALS, suggesting that the proposed method is robust and that neither region presents differential risk or protective factors.

## Introduction

Motor neuron diseases (MND) are a group of mainly sporadic neurodegenerative diseases characterised by a progressive loss of upper and/or lower motor neurons (UMN and LMN, respectively) that frequently leads to progressive paralysis and death, typically due to respiratory failure^1^. Based on the degree of clinical and neurophysiological impairment of the UMN and LMN, MNDs are further divided into three major diagnostic categories: amyotrophic lateral sclerosis (ALS)—by far the most frequent MND comprising about 90% of MND patients—and the related diseases, primary lateral sclerosis (PLS) and progressive muscular atrophy (PMA). Despite sharing causes, pathology and pathophysiological mechanisms, their different prognoses and differential diagnoses justify their distinction in both clinical practice and research^1^.

In clinical practice, different phenotypes of ALS can be distinguished based on the site of onset (i.e. bulbar, spinal, respiratory), the presence and degree of cognitive impairment (i.e. ALS with frontotemporal dementia or ALS-FTD), or the presence of causal mutations (i.e. familial vs sporadic ALS), among others^1^.

PLS is characterised by a pure UMN involvement for at least four years, with a slow progression, typically with long-term stationary phases and frequently normal lifespan^2^. The period of four years for a definitive PLS diagnosis is required because LMN signs can develop in this time, and the patient is subsequently reclassified into the ALS category^3,4^.

PMA is characterised by a pure clinical impairment of the LMN^5^. Unlike PLS, there is no established definition for PMA, yet the distinction is considered useful for its prognostic implications, and a similar 4-year period has been proposed to distinguish it from the ALS forms with predominantly LMN impairment^1^.

### Prevalence and incidence of ALS

Recent population-based studies have reported a prevalence of ALS of between 4.1 and 8.4 per 100,000 inhabitants, with a pooled prevalence of about 4.4 per 100,000 inhabitants^6–11^.

Prevalence estimates show a marked geographical heterogeneity. In the USA, prevalences of 5 per 100,000 have been reported for ALS patients^12–14^. Furthermore, from the work carried out within the 'Global Burden of Disease', also in the USA, an age-standardized prevalence of motor neuron disease was estimated as being equal to 9.1 per 100,000 in 2007^15^. Meanwhile, Zapata et al. estimated a point prevalence of 4.9 per 100,000 inhabitants) for Antioquia, Colombia in 2014^16^ and by combining 13 studies, Chiò et al. estimated a median crude prevalence of 5.4 per 100,000 for Europe, ranging from 1.1 in Yugoslavia, to 8 in the Netherlands and two Italian regions^9^, and 8.2 in the Faroe Islands^9,17^. In Asia, the prevalence is usually lower than in Western European countries^11^. The median crude prevalence has been estimated at 1.0 per 100,000 in China^9^ and 1.57 for South Asia^11^. On the other hand, the prevalence in Oceania and Japan are much higher^11^.

Recent studies have reported a global incidence of ALS of between 0.6 and 3.8 per 100,000 person-year, with a pooled worldwide incidence of about 1.6–1.7^6,11,18^.

In Europe the incidence is higher, ranging from 1.5 to 3.8 per 100.000 person-years in the decade of the 1990s^19^ and with a median incidence of 2.08 per 100,000 person-years^1,6,11,17^. Lower incidences are found in Southern Europe compared with Western and Northern Europe^18^.

While incidence is usually much lower in Asia than it is in Europe, there is considerable variability among countries. The standardised incidence is about 0.83 per 100,000 person-years for East Asia^6,28^, and 0.73 per 100,000 person-years for South Asia^18^. For the same period in Japan, however, the incidence of ALS was estimated at 2.2 per 100,000 person-years^20^ and 1.2 per 100,000 person-years in South Korea^6^.

With respect to the Americas, the Global Burden of Disease estimated an age-standardized incidence for motor-neuron disease equal to 1.9 per 100,000 inhabitants for the USA in 2007^15^, while Zapata et al*.* estimated an incidence of 1.4 per 100,000 person-years for Antioquia, Colombia^16^.

Heterogeneity in both the incidence and prevalence of ALS is also present when other variables such as age and sex are considered. For example, incidence is between 1.3 and 1.5 times higher in men than in women^7,9,11,13,17,18,21–24^ and it is also known that ALS is very rare prior to 40 years of age. The incidence of ALS reaches its maximum between 60 and 75 years^9,22–24^ or between 65 and 85 years^11,17^, depending on the study population.

To the best of our knowledge, no previous studies have analysed the prevalence or incidence of PLS and PMA. This may be because they are very rare diseases with a challenging differential diagnosis, thus making it difficult to carry out population-based studies.

The aim of this study was to estimate the prevalence and incidence of MND and its different diagnostic categories (ALS, PLS, and PMA), in the regions of Catalonia and Valencia (Spain) in the period 2011–2019.

## Materials and methods

### Design

**Figure 1:**
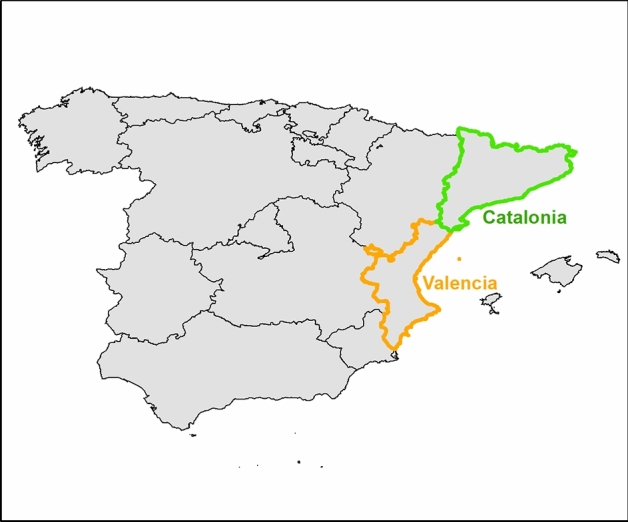
Location of the Catalonia and Valencia regions.

Two Spanish Mediterranean regions were analysed, Catalonia and Valencia, which together comprise 26.72% of the total Spanish population and 53.67% of the Spanish population living in the Mediterranean basin (Catalonia 7,566,000 inhabitants and Valencia 4,975,000 inhabitants)^25^ (Fig. 1). These two neighbouring regions share climatic and sociodemographic conditions and have a common history (both belonged to the Kingdom of Aragon, one of the two kingdoms comprising Spain from the Middle Ages onwards), and much of the Catalan population originates from Valencia and vice-versa. Thus, it is likely that these populations have shared genetic and environmental risk factors of MND.

The study population was made up of the residents in the regions of Catalonia and Valencia between 1 January 2011 and 31 December 2019 (Catalonia) and between 1 January 2013 and 31 December 2018 (Valencia).

Two population-based cohorts were used, (one from Catalonia and the other from Valencia), encompassing the entire population of both regions. In both cohorts, clinical data were prospectively obtained, while some demographic data were retrospectively collected. The cohorts included all the patients who had been assessed at two Motor Neuron Disease Functional Units (MNDFU) in Spain: the Bellvitge University Hospital in Hospitalet de Llobregat in Catalonia, and the La Fe University and Polytechnic Hospital in Valencia, which act as referral units in the Catalan and Valencian regions, respectively. All the patients with MND seen in the Bellvitge Hospital since 2011 and the La Fe Hospital since 2013 were recorded prospectively in a database created for this purpose, and which included their demographic and clinical characteristics. All the patients studied were adults (specifically, the minimum age was 21 years in the Valencia cohort and 25 in the Catalonia cohort).

This study included the patients who had been evaluated in either of the two referral units during the time periods stated above and who met the following criteria: (1) a diagnosis of ALS, defined as the presence of UMN and LMN dysfunction in at least one body region at any time of the disease course, or LMN dysfunction in at least two body regions with a disease course typical of ALS (continuous non-invasive ventilation or death within four years from disease onset)^26^; (2) a diagnosis of definite PLS according to recent criteria^3^; (3) PMA diagnosis, considered as the presence of isolated signs of LMN in at least two body regions with a slowly progressing disease course and a prolonged survival (> 4 years). Although there is no agreed consensus about the definition of PMA, to be consistent with the definition used for PLS, the recently proposed four-year criteria from Al-Chalabi et al.^1^ was used. The diagnosis of MND and its different diagnostic categories (ALS, PLS and PMA) was made by specialists in MND.

All the census tracts of all the municipalities in Catalonia and Valencia were included; some with cases of MND and others (a much higher number) with no cases.

### Variables

#### Response variable

Different models were estimated depending on the response variable. First, all MND cases were considered as the response variable, regardless of diagnostic category. Second, the following diagnostic categories were considered as additional response variables: ALS, PLS and. PMA. This was done separately for each region (Catalonia and Valencia).

#### Individual control variables

Age at diagnosis and sex were included as individual explanatory variables. Age was further categorised into quintiles, taking the first quintile as the reference category. For the sex variable, male was considered as the reference category.

Patients were further categorized, according to the symptom of onset, into spinal, bulbar, respiratory and cognitive. The *C9ORF72* expansion was studied in all MND patients, whereas *SOD1* was sequenced in those familial MND patients not carrying the *C9ORF72* expansion. In PMA patients, expansions in the androgen receptor gene were appropriately excluded.

#### Contextual control variables

The Spanish Epidemiology Association’s Deprivation Index 2011 (IP2011)^27^ corresponding to each of the census tracts of the municipalities of each of the two regions, was included as a contextual explanatory variable of interest. The IP2011 combines the information on six socioeconomic indicators, calculated for each census tract and based on the data collected in the Spanish Population and Housing Census 2011^28^: percentage of the manual worker population, percentage of the temporary working population, percentage of the unemployed population, percentage of the population without complete compulsory schooling, and percentage of primary residences without access to the Internet The IP2011 was considered as the first component of a sequential principal component analysis (PCA). First, a PCA with ten selected indicators was carried out (in addition to the six already mentioned, the following were included: the percentage of the population born in low-income countries and arrived in Spain after 2006, the percentage of the population born in low-income countries or born in Spain whose father or mother were born in low-income countries, the percentage of the population aged 65 or over, and the percentage of single-parent households with a woman in charge). Second, the ten indicators were correlated (using Spearman's correlation coefficient) with the first component obtained through the PCA. Third, those indicators with correlations less than 0.5 were excluded. Finally, a PCA was carried out using the six indicators finally considered (further information can be found in^27^). The deprivation index was categorised into quintiles, taking the first quintile (i.e. the one corresponding to the less economically deprived census tracts) as the reference category.

The populations in each of the two regions, stratified by sex and age groups (women under 16 years, women aged 16 to 64 years, women ≥ 65 years, men under 16 years, men aged 16 to 64 years, men ≥ 65 years), were also included as contextual explanatory variables. The reference categories were the youngest categories for each sex. The total population at risk (population aged over 16 years) was used as the offset in the model for each of the two regions.

Population data by census tract, age, and sex was obtained from the Spanish Population and Housing Census 2011^28^.

The graphic representation was produced based on the cartography of Catalonia (SRC ED50/UTM zone 31 N) and Valencia (SRC ED50/UTM zone 30 N) at the census tract level for 2011^29^.

### Statistical analysis

Selection bias is common in observational designs. That is, the analysed cohorts do not constitute a random sample of the population. Some subjects have a higher probability of being observed than others and, therefore, of being present in the sample. To this effect, the subjects that come into contact with one of the referral MNDFUs in the regions studied will be overrepresented.

If the selection was exogenous (also termed ignorable non-response, missing at random, or selection on observables), or in other words if the probability of a subject to be observed was identical for all subjects, weighting the sample to give less weight to the subjects actually observed would be sufficient. The weighting used would be the same as the inverse of the percentage of subjects among the population seen at each hospital, stratified by sex and age group. In epidemiology, this method of weighting is called standardisation by age and sex^30^, considering the population covered by each hospital as the standard population.

However, these subjects are very likely to go to one of the referral MNDFUs, not only because they have symptoms of the onset of the disease, but also because there are unobserved factors that influence their use, which would be correlated with the non-observable factors that affect the response variable^31^. In this sense, the population served by the MNDFU has a centre bias. In other words, they do not serve all the patients in the respective populations, but rather a part that may be biased (e.g. some centres send more patients than others; the patients they send are younger than the real population, etc.). In all cases, the probability of these subjects being observed is not the same for all the subjects. Therefore, weighting (standardisation) by age and sex would not correct the selection bias^31^.

In this case (known as endogenous selection, also termed informative selection, missing not at random, or selection on unobservables), other more complex statistical methods should be used to obtain unbiased estimates of prevalence and incidence, something that standardization by age and sex does not permit. Therefore, we chose a two-part model^32^. In the first part, the probability of a subject being observed is estimated. These probabilities are then used as weightings in the second part of the model, where the prevalence and incidence are estimated, with the aim of correcting the non-randomness (in other words, the selection bias). The two parts are typically estimated using a two-part model called Hurdle^32,33^. Thus, we specified a Hurdle model in which the two components were estimated jointly^33^.

In the first part, the probability that an individual is being observed is modelled using a generalised linear mixed model (GLMM) with a binomial link. Included as variables associated with this probability are those that could explain that a subject was observed (in other words, that he/she had had contact with the UFMN of the corresponding hospital). More specifically, we considered individual and contextual variables.

Conditional on the true risk at the location ${x}_{i}$, the probability of a case (of the corresponding response variable) occurring in this location, $P\left({x}_{i}\right)$, $i=1,\dots , n$, is distributed as a binomial.

$${Y}_{i}\left|P\left({x}_{i}\right)\right.\sim Binomial\left({n.trials}_{i},P\left({x}_{i}\right)\right)$$

${n.trials}_{i}$ is the population at risk of being a case in the location ${x}_{i}$.

The link function is as follows:

$$\mathrm{log}\left(\frac{P\left({x}_{i}\right)}{1-P\left({x}_{i}\right)}\right)={\beta }_{0}+{\eta }_{1i}+S\left({x}_{i}\right)+{\beta }_{1} {gender}_{i}+\sum_{k=2}^{5}{\beta }_{2k} {age\_diagQ}_{ik}+\sum_{k=2}^{5}{\beta }_{3k} {deprivationQ}_{ik}+{\beta }_{4} log\left({men\_16to64}_{i}\right)+{\beta }_{5} log\left({men\_65ormore}_{i}\right)+{\beta }_{6} log\left({women\_16to64}_{i}\right)+{\beta }_{7} log\left({women\_65ormore}_{i}\right)+offset\left({population\_over16}_{i}\right)$$

where the subindex $i$ indicates the census tract of the municipalities in each of the two regions and $\beta$ are the coefficients of the explanatory variables (${e}^{\beta }$ is the relative risk associated with each of them). $\eta$ and $S$ are random effects. $\eta$ indicates the spatially unstructured individual heterogeneity. In other words, it indicates the non-observable confounders associated with every individual which do not vary over time. $S$ is a spatially structured random effect normally distributed with zero mean and a Mátérn covariance function:

$$Cov\left(S\left({x}_{i}\right),S\left({x}_{{i}^{^{\prime}}}\right)\right)=\frac{{\sigma }^{2}}{{2}^{\nu -1}\Gamma \left(\nu \right)} {\left(\upkappa \Vert {x}_{i}-{x}_{{i}^{^{\prime}}}\Vert \right)}^{\nu } {\mathrm{\rm K}}_{\nu } \left(\upkappa \Vert {x}_{i}-{x}_{{i}^{^{\prime}}}\Vert \right)$$

where ${\mathrm{\rm K}}_{\nu }$ is the modified Bessel function of the second type and order $\nu >0$. $\nu$ is a smoother parameter, ${\sigma }^{2}$ is the variance and $\kappa >0$ is related to the range ($\rho =\sqrt{8 \nu }/\kappa$), the distance to which the spatial correlation is close to 0.1.

Because of the large number of census tracts with no cases, in the second part a GLMM with Zero Inflated Poisson distribution is used to model the number of patients diagnosed for each of the response variables and by census tract. The same explanatory variables as in the first part of the model are included.

Conditional to the true risk in the location ${x}_{i}$, the mathematic expectation of cases of the response variable occurring in each of the census tracts, $\theta \left({x}_{i}\right)$, $i=1,\dots , n$, is distributed as a Poisson:

$${Y}_{i}\left|{\theta }_{i}\right.\sim Poisson\left({\theta }_{i}\right)$$

In this case, the link function is as follows:

$$\mathrm{log}\left({\theta }_{i}\right)={\beta }_{0}+{\eta }_{1i}+S\left({x}_{i}\right)+{\beta }_{1} {gender}_{i}+\sum_{k=2}^{5}{\beta }_{2k} {age\_diagQ}_{ik}+\sum_{k=2}^{5}{\beta }_{3k} {deprivationQ}_{ik}+{\beta }_{4} log\left({men\_16to64}_{i}\right)+{\beta }_{5} log\left({men\_65ormore}_{i}\right)+{\beta }_{6} log\left({women\_16to64}_{i}\right)+{\beta }_{7} log\left({women\_65ormore}_{i}\right)+offset\left({population\_over16}_{i}\right)$$

The definition of the parameters, the variables, and the random effects, is the same as described previously.

In both parts, random effects that include non-observed confounders are also included, capturing individual heterogeneity (specific for each of the two parts) and spatial dependence. That is to say, areas that are close in space show more similar prevalence and incidence than areas that are not close (common to the two parts). In the model used to estimate the incidence, an additional random effect was included to control for the existence of temporal trends (also common to the two parts). The temporal dependence was controlled using a random effect structured as a random walk of order 1^34^ (further details can be found elsewhere^33^).

Both parts were estimated simultaneously following a Bayesian perspective, using the Integrated Nested Laplace Approximation (INLA) approach^35^. Priors that penalize the complexity (PC priors)^36^ and have been found to be very robust were used.

Consequently, point and interval estimates were obtained for the prevalence and the incidence, both at the level of the region to which the hospital belongs, and at the level of census tract of each of their municipalities.

Last, the prevalence and incidence of MND per 100,000 inhabitants is represented on the map of the census tracts of Catalonia and Valencia using the mapping by census tracts of the Population and Housing Census 2011^28^, and the “leaflet” library^37^ (Figs. 3 and 4).

All analyses were carried out using the free software R (version 3.6.2)^38^, through the INLA package^35,39^.

### Ethics approval

The data for this study came from an anonymised clinical administrative database and only the lead researcher, where necessary, had access to the identity of each individual. The information in this administrative clinical database was obtained through the informed consent of the patients. In any case, all methods of this study were carried out in accordance with relevant guidelines and regulations, having been revised and approved by the Committee of Ethics and Clinical Research (CEIC) of the Bellvitge University Hospital.

## Results

Between 2013 and 2018, 259 MND patients were recorded in the Valencian MNDFU, whereas 591 cases were recorded in the Catalonian MNDFU between 2013 and 2019. Their demographic and clinical characteristics can be found in Tables 1, 2 and 3. In most of the census tracts (93.0% of the census tracts for Valencia, and 89.4% for Catalonia) no cases of MND were found (Table 4). 6.6% and 9.6% of the census tracts of Valencia and Catalonia, respectively, had just one case of MND. The maximum number of cases of MND in a census tract was three. However, although the populational structure is very similar in Catalonia and Valencia (the percentage of the population aged 65 years and over was 16.70% in Catalonia and 16.90% in Valencia), the census tracts from the municipalities of Valencia were much more deprived than those in Catalonia (average of 0.082 in Valencia and − 0.470 in Catalonia, median of 0.110 in Valencia and − 0.503 in Catalonia).

**Table 1:** Descriptive analysis of demographic and clinical characteristics by region for each of the diagnostic categories. Amyotrophic Lateral Sclerosis (ALS).

Demographic and clinical characteristics stratified by region		
	Valencia (n = 219)	Catalonia (n = 524)
**Variables**		
**Sex [n (%)]**		
Women	99 (45.2)	246 (46.9)
Men	120 (54.8)	278 (53.1)
Age at diagnosis^a^	62.79 (11.30)	63.68 (12.47)
	63 (56.0–70.0)	65.0 (56.0–73.0)
	(21–86)	(25–89)
**Age at diagnosis in quintiles [n (%)]**		
< = 53/ < = 52	35 (16.0)	92 (17.6)
(53–59]/(52–61]	50 (22.8)	105 (20.0)
(59–66]/(61,67]	41 (18.7)	109 (20.8)
(66–71]/(67,74.6]	48 (21.9)	105 (20.0)
> 71/ > 74.6	45 (20.5)	113 (21.6)
**Symptom at onset [n (%)]**		
Bulbar	62 (28.31)	155 (29.6) (n = 512)
Spinal	146 (66.67)	324 (61.8) (n = 512)
Respiratory	4 (1.83)	20 (3.8) (n = 512)
Cognitive (dementia)	7 (3.20)	13 (2.5) (n = 512)
C9orf72 [n (%)]	17 (8.25) (n = 206)	33 (8.82) (n = 374)
SOD1 [n (%)]	5 (2.43) (n = 206)	2 (1.87) (n = 107)

^a^First row: mean (sd).

Second row: median (1st–3rd quartiles).

Third row: (min–max).

**Table 2:** Descriptive analysis of demographic and clinical characteristics by region for each of the diagnostic categories. Progressive Muscular Atrophy (PMA)

Demographic and clinical characteristics stratified by region		
	Valencia (n = 27)	Catalonia (n = 14)
Variables		
**Sex [n (%)]**		
Women	5 (18.5)	2 (14.3)
Men	22 (81.5)	12 (85.7)
Age at diagnosis^a^	60.70 (14.28)	55.50 (13.33)
	64 (52.0–72.0)	54.0 (42.8–68.0)
	(23–78)	(36–76)
**Age at diagnosis in quintiles [n (%)]**		
< = 53/ < = 52	7 (25.9)	7 (50.0)
(53–59]/(52–61]	3 (11.1)	1 (7.1)
(59–66]/(61,67]	5 (18.5)	2 (14.3)
(66–71]/(67,74.6]	5 (18.5)	3 (21.4)
> 71/ > 74.6	7 (25.9)	1 (7.1)
**Symptom at onset [n (%)]**		
Bulbar	2 (7.4)	0 (0.0) (n = 6)
Spinal	25 (92.6)	6 (100.0) (n = 6)
Respiratory	0 (0.0)	0 (0.0) (n = 6)
Cognitive (dementia)	0 (0.0)	0 (0.0) (n = 6)
C9orf72 [n (%)]	0 (0.0) (n = 26)	0 (0.0) (n = 13)
SOD1 [n (%)]	3 (11.54) (n = 26)	0 (0.0) (n = 4)

^a^First row: mean (sd).

Second row: median (1st-3rd quartiles).

Third row: (min–max).

**Table 3:** Descriptive analysis of demographic and clinical characteristics by region for each of the diagnostic categories. Primary Lateral Sclerosis (PLS).

Demographic and clinical characteristics stratified by region		
	Valencia (n = 10)	Catalonia (n = 4)
Variables		
**Sex [n (%)]**		
Women	5 (50.0)	3 (75.0)
Men	5 (50.0)	1 (25.0)
Age at diagnosis^a^	59.20 (12.48)	56.00 (12.52)
	60 (55.75–68.25)	60.5 (42.75–64.75)
	(28–73)	(38–65)
**Age at diagnosis in quintiles [n (%)]**		
< = 53/ < = 52	1 (10.0)	1 (25.0)
(53–59]/(52–61]	3 (30.0)	1 (25.0)
(59–66]/(61,67]	3 (30.0)	2 (50.0)
(66–71]/(67,74.6]	2 (20.0)	0 (0.0)
> 71/ > 74.6	1 (10.0)	0 (0.0)
**Symptom at onset [n (%)]**		
Bulbar	3 (30.0)	NA
Spinal	7 (70.0)	NA
Respiratory	0 (0.0)	NA
Cognitive (dementia)	0 (0.0)	NA
C9orf72 [n (%)]	0 (0.0)	0 (0.0) (n = 3)
SOD1 [n (%)]	0 (0.0)	0 (0.0) (n = 0) No test available

^a^First row: mean (sd).

Second row: median (1st–3rd quartiles).

Third row: (min–max).

**Table 4:** Description of the contextual variables by census tract.

Description of the contextual variables by por census tract				
		Valencia (n = 259)		Catalonia (n = 591)
Variables				
Census tracts		3,473		5,018
**Cases MND by tract [n (%)]**				
0		3,229 (93.0)		4,485 (89.4)
1		230 (6.6)		481 (9.6)
2		13 (0.4)		46 (0.9)
3		1 (0.0)		6 (0.1)
**Cases ALS by tract [n (%)]**				
0		3,264 (94.0)		4,538 (90.4)
1		199 (5.7)		440 (8.8)
2		10 (0.3)		37 (0.7)
3		0 (0.0)		3 (0.1)
**Cases PMA by tract [n (%)]**				
0		3,447 (99.3)		5,004 (99.7)
1		25 (8.4)		14 (0.3)
2		1 (0.0)		0 (0.0)
**Cases PLS by tract [n (%)]**				
0		3,463 (99.7)		5,014 (99.9)
1		10 (0.3)		4 (0.1)
Total population [n]		4,990,470		7,472,920
Population under 16 years		808,805		1,246,595 (missing = 64)
Population over 16 years		4,180,520		6,226,325
Population 16 to 64 years		3,336,200		4,978,106
Population 65 years and over		843,265 (missing = 14)		1,248,219
Men [n]		2,481,257		3,701,280
Men under 16 years		416,825 (missing = 17)		645,025 (missing = 114)
Men over 16 years		2,060,847 (missing = 17)		3,056,415
Men 16 to 64		1,693,039		2,520,051 (missing = 12)
Men 65 years and over		370,258 (missing = 33)		531,909 (missing = 72)
Women [n]		2,508,893		3,771,595
Women under 16 years		391,980 (missing = 17)		601,570 (missing = 122)
Women over 16 years		2,114,278 (missing = 15)		3,170,035
Women 16 to 64		1,643,225		2,456,554 (missing = 15)
Women 65 years and over		473,083 (missing = 31)		707,914 (missing = 74)
Deprivation index Mean (standard deviation)		0.082 (0.862)		− 0.470 (0.784)
Median (Q1–Q3)		0.110 (− 0.423–0.601)		− 0.503 (− 1.015–0.005)

*MND* moto neuron disease, *ALS* amyotrophic lateral sclerosis, *PMA* progressive muscular atrophy, *PLS* primary lateral sclerosis.

Table 5 shows the main demographic and clinical characteristics of patients diagnosed with MND, stratified by region. Mean age at diagnosis was 62.43 (median = 63) years in Valencia and 63.51 (median = 65) years in Catalonia. In both regions, there were more men than women (57.9% in Valencia and 55.5% in Catalonia). When the diagnostic category is taken into account, 85.5% of all MND cases in Valencia were ALS, 10.5% were PMA, and 3.9% PLS. The same pattern was observed for Catalonia, with ALS being the most frequent diagnosis (96.7%), followed by PMA (2.6%) and PLS (0.7%). Among ALS patients in Valencia, 66.67% had spinal ALS, 28.31% bulbar ALS, 3.20% cognitive onset, and 1.83% respiratory ALS. Similar patterns were seen in Catalonia: 64.12% spinal ALS, 29.58% bulbar ALS, 3.82% respiratory ALS, and 2.48% cognitive onset. Finally, in Valencia 6.95% of the patients carried a *C9ORF72* expansion and 3.27% the *SOD1* mutation, while in Catalonia it was 7.67% and 2.22%, respectively.

**Table 5:** Descriptive analysis of demographic and clinical characteristics by region. All patients.

Demographic and clinical characteristics stratified by region		
	Valencia (n = 259)	Catalonia (n = 591)
Variables		
**Sex [n (%)]**		
Women	109 (42.1)	263 (44.5)
Men	150 (57.9)	328 (55.5)
Age et al. diagnosis^a^	62.43 (11.67)	63.51 (12.54)
	63 (56.0–70.0)	65.0 (56.0–73.0)
	(21–86)	(25–89)
**Age at diagnosis in quintiles [n (%)]**		
< = 53/ < = 52	45 (17.4)	106 (17.9)
(53–59]/(52–61]	57 (22.0)	117 (19.8)
(59–66]/(61,67]	49 (18.9)	122 (20.6)
(66–71]/(67,74.6]	55 (21.2)	121 (20.5)
> 71/ > 74.6	53 (20.5)	125 (21.2)
**Definite diagnosis [n (%)]**		
ALS	219 (85.5)	524 (96.7)
PMA	27 (10.5)	14 (2.6)
PLS	10 (3.9)	4 (0.7)
**Symptom at onset [n (%)]**		
Bulbar	62 (28.31)	155 (29.58)
Spinal	146 (66.67)	336 (64.12)
Respiratory	4 (1.83)	20 (3.82)
Cognitive (dementia)	7 (3.20)	13 (2.48)
C9orf72 [n (%)]	17 (6.94) (n = 245)	33 (7.67) (n = 430)
SOD1 [n (%)]	8 (3.27) (n = 245)	3 (2.22) (n = 135)

*ALS* Amyotrophic Lateral Sclerosis, *PMA* Progressive Muscular Atrophy, *PLS* Primary Lateral Sclerosis.

^a^First row: mean (sd).

Second row: median (1st-3rd quartiles).

Third row: (min–max).

**Figure 2:**
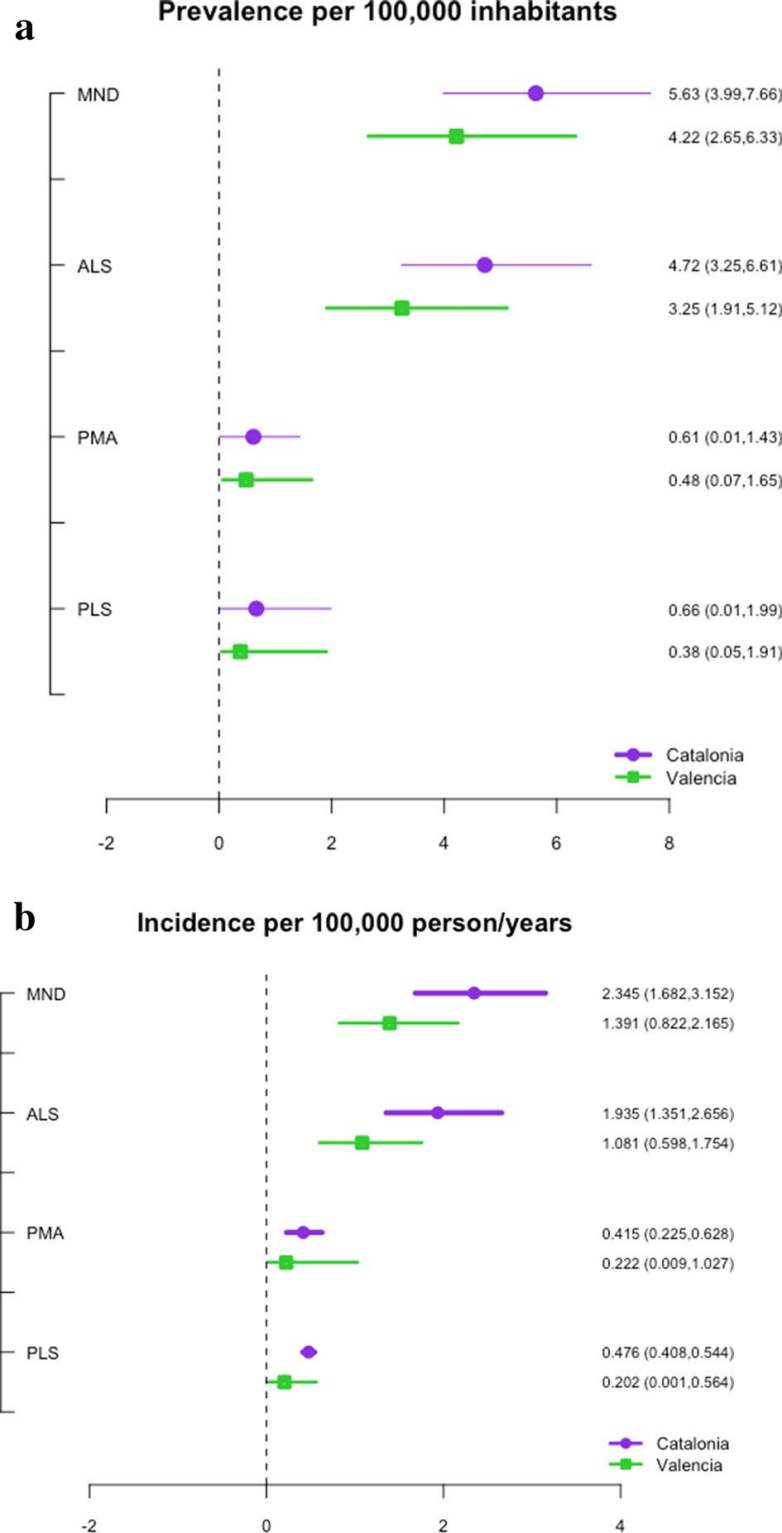
(**a**) Forest plot of the estimated prevalence per 100,000 inhabitants in Catalonia and Valencia. (**b**) Forest plot of the estimated incidence per 100,000 person-years in Catalonia and Valencia.

Table 6 shows the estimated prevalence and incidence of MND (point estimation and credibility interval at 95%), for the two regions, as well as by diagnostic category, per 100,000 inhabitants. Although the point estimator of MND prevalence was higher for Catalonia than for Valencia (5.627 vs 4.216, respectively), there were no statistically significant differences between the two regions, given that the credibility intervals overlapped (shown graphically in the forest plot in Fig. 2a). Likewise, although the prevalence of ALS, PMA and PLS observed in Catalonia were always above those of Valencia (ALS 4.72 vs 3.25, PMA 0.61 vs 0.48, PLS 0.66 vs 0.38, respectively), the differences between the two regions were not statistically significant.

**Table 6:** Estimation of the prevalence and incidence of motor neuron disease (MND) and its variants in the regions of Valencia and Catalonia (2013–2018 and 2011–2019).

Estimation of the prevalence and incidence of mnd and variants, stratified by region			
		Valencia (n = 259)	Catalonia (n = 591)
Prevalence (per 100,000 inhabitants)			
**Variables**			
MND^a^		4.216 (2.652–6.334)	5.627 (3.990–7.662)
**Definite diagnosis**^**a**^			
ALS		3.249 (1.911–5.120)	4.721 (3.248–6.608)
PMA		0.480 (0.065–1.650)	0.602 (0.008–0.634)
PLS		0.378 (0.046–1.896)	0.662 (0.000–1.987)
Incidence (per 100,000 person-years)			
**Variables**			
MND^a^		1.390 (0.822–2.165)	2.346 (1.682–3.152)
**Definite diagnosis**^**a**^			
ALS		1.080 (0.598–1.754)	1.935 (1.351–2.656)
PMA		0.222 (0.009–1.027)	0.415 (0.225–0.628)
PLS		0.202 (1.306e-09–0.564)	0.476 (0.409–0.544)

*MND* Moto Neuron Disease, *ALS* Amyotrophic Lateral Sclerosis, *PMA* Progressive Muscular Atrophy, *PLS* Primary Lateral Sclerosis.

^a^Point estimate (credibility interval at 95%).

Regarding the estimation of the incidence of MND and their variants by region, Table 6 and Fig. 2b show a greater incidence in Catalonia than in Valencia: MND (2.346 vs 1.390 per 100,000 person-year, respectively), ALS (4.721 vs 3.249 per 100,000 person-year, respectively), PMA (0.415 vs 0.222 per 100,000 person-year, respectively) and PLS (0.476 vs 0.202 per 100,000 person-year, respectively). However, differences were not statistically significant, given that the credibility intervals overlapped in the two cases.

**Figure 3:**
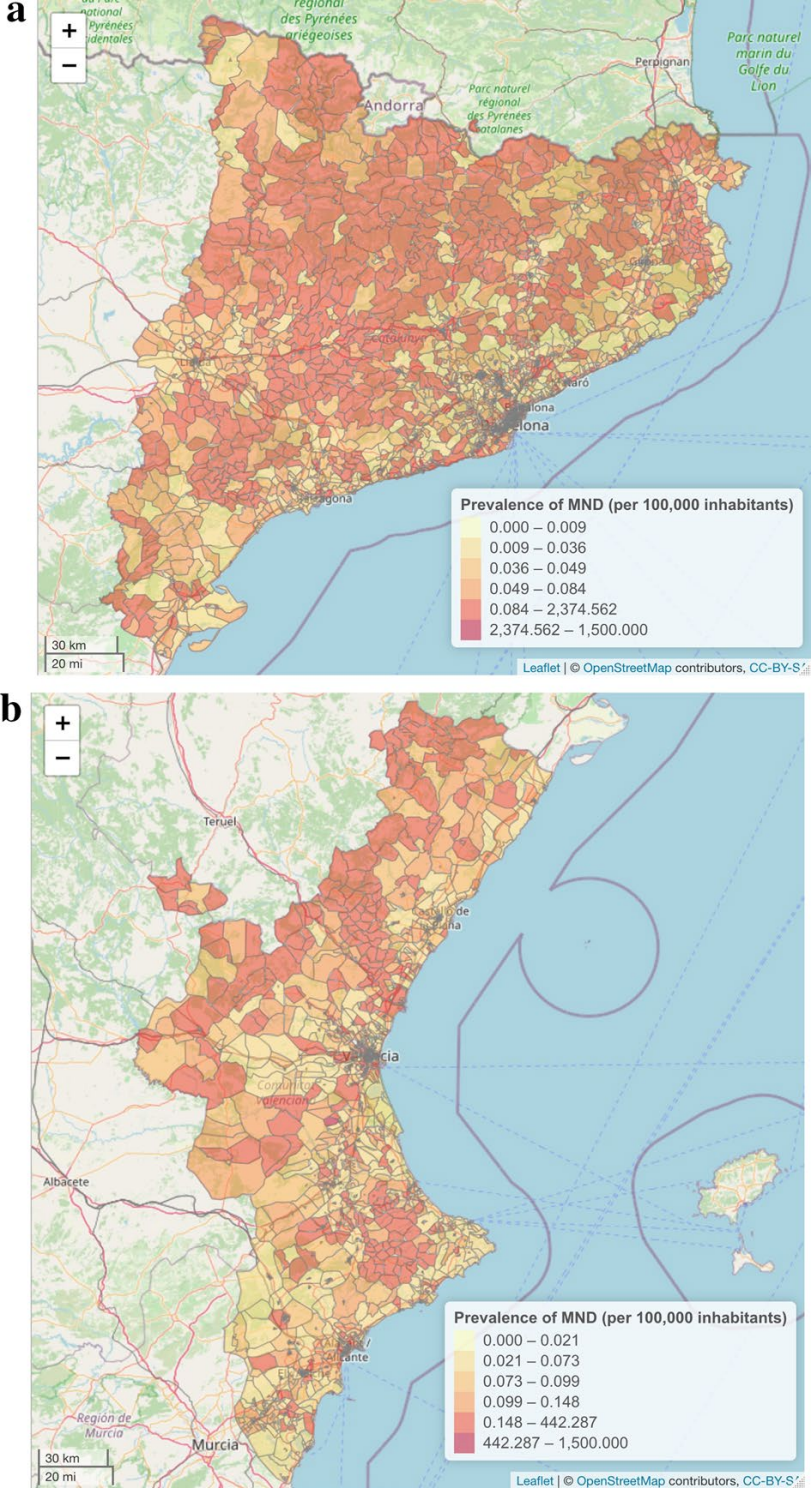
(**a**) Map of the estimated prevalence per census tract across the study region (Catalonia). Own construction using the leaflet package^37^, version 2.0.3 [https://CRAN.R-project.org/package=leaflet]. (**b**) Map of the estimated prevalence per census tract across the study region (Valencia). Own construction using the leaflet package^37^, version 2.0.3 [https://CRAN.R-project.org/package=leaflet].

**Figure 4:**
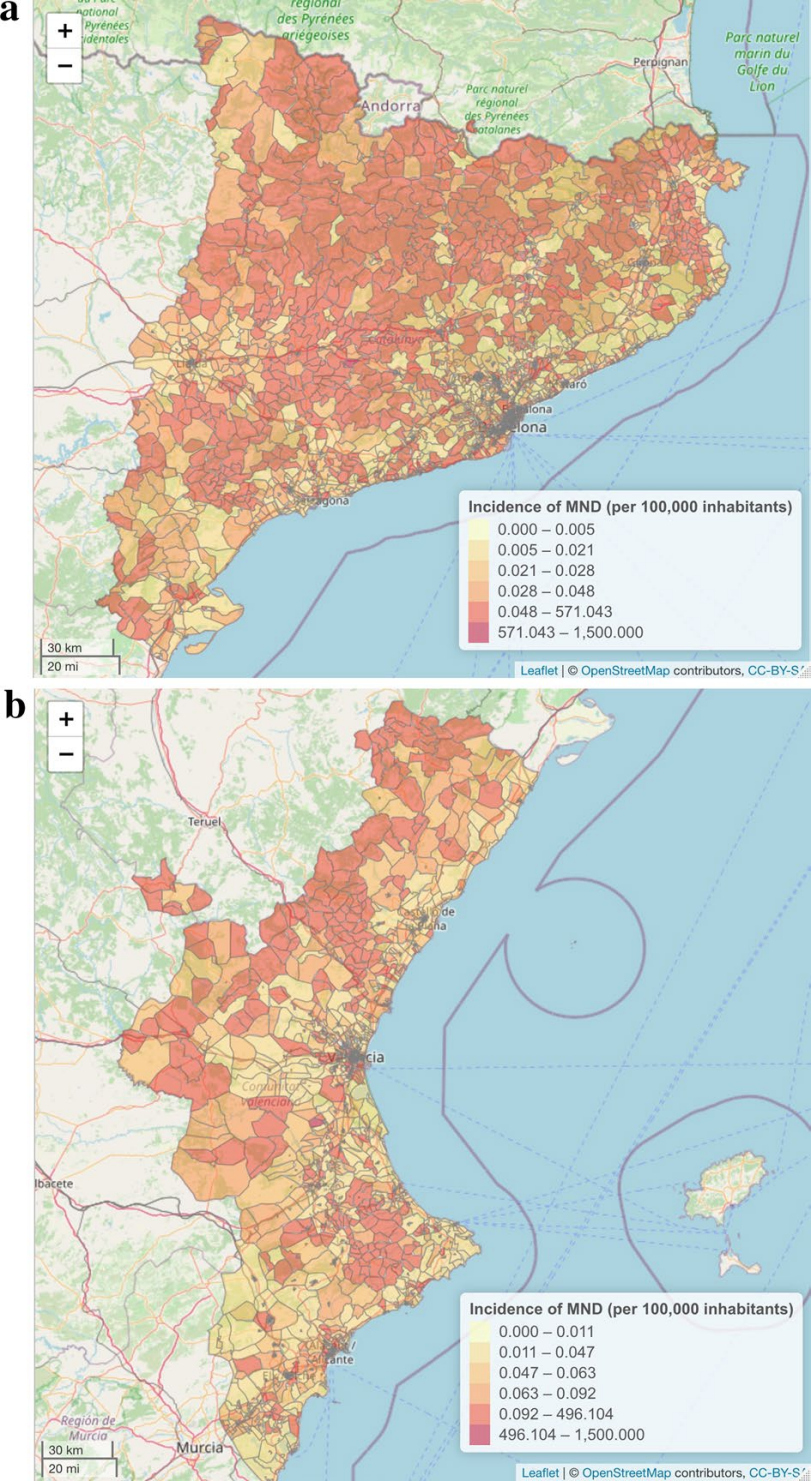
(**a**) Map of the estimated incidence per census tract across the study region (Catalonia). Own construction using the leaflet package^37^, version 2.0.3 [https://CRAN.R-project.org/package=leaflet]. (**b**) Map of the estimated incidence per census tract across the study region (Valencia). Own construction using the leaflet package^37^, version 2.0.3 [https://CRAN.R-project.org/package=leaflet].

Finally, Fig. 3a,b show the estimated prevalence by census tract in Catalonia and Valencia, and Fig. 4a,b are a graphic representation of the estimated incidence by census tract. The census tracts whose at risk populations are very small must be interpreted with caution.

In summary, and using the intersection regions of the credibility intervals of the two regions, it was estimated with a 95% probability for the period 2011–2019 (Catalonia 2011–2019, Valencia 2013–2018) that the prevalence of MND would be between 3.990 and 6.334 per 100,000 inhabitants; the prevalence of ALS between 3.248 and 5.120 per 100,000 inhabitants; the prevalence of PMA between 0.065 and 0.634 per 100,000 inhabitants; and the prevalence of PLS between 0.046 and 1.896 per 100,000 inhabitants.

Regarding incidence, again with a 95% probability and for the period 2011–2019 (Catalonia 2011–2019, Valencia 2013–2018), it was estimated as being between 1.682 and 2.165 per 100,000 person/years for MND; between 1.351 and 1.754 per 100,000 person/years for ALS; between 0.225 and 0.628 per 100,000 person/years for PMA; and between 0.409 and 0.544 per 100,000 person/years for PLS.

## Discussion

This work makes a number of important contributions. First, it provides prevalence and incidence estimators not only of MND itself, but also of ALS, PLS and PMA. Second, an original prevalence and incidence estimation method is proposed, that controls the selection bias caused by not using random samples from the population. Third, to meet the objective of this study, both individual and contextual databases from various and different sources are needed, which is where Real-World Data takes on importance. This approach is used in this work to combine the individual-level databases with a contextual-level database containing population data and data on the socioeconomic level of the area where the subjects live. Fourth, the existence of non-observed confounders and the spatial and temporal dependence inherent to the data used are also controlled for and last but not least, the study compares the prevalence and incidence in two different Spanish regions, allowing for any differential risk or protective factors in them to be assessed.

### Characteristics of the patients

Although the cohorts studied were not random samples from the population, the demographic and clinical characteristics of the ALS patients (Table 1) are almost identical to those of previous population-based studies in the Mediterranean area^40^, suggesting that our cohorts were representative of the ALS populations in Valencia and Catalonia, respectively. Moreover, the frequency of the C9ORF72 expansion in both ALS cohorts (8.2–8.8%) was very similar to that previously found in a Spanish study (7.2%)^41^. The frequency of the SOD1 mutation (1.9–2.4%) was somewhat lower than that previously reported (4.25%)^42^, probably because, unlike C9ORF72, SOD1 is not routinely studied in sporadic ALS patients.

As expected^2^, PLS represented approximately 3% of MND patients and their characteristics were similar to previously described cohorts of PLS patients. Specifically, they were younger than the ALS patients (Tables 2 and 3). PMA data must be compared with previous reports with caution, since there has previously been no uniform definition of PMA. For this study, the recently proposed definition of ‘restricted LMN signs for at least four years’^1^ was used to clearly differentiate these patients from LMN-predominant ALS patients. Despite possible differences in the definition, the characteristics of the PMA patients were similar to previous reports^43,44^: a frequency of 2.6–10.5% of MND patients, a striking male and spinal-onset predominance, and a similar age to that of ALS patients. Importantly, in the PMA patients’ expansions in the androgen receptor gene had been appropriately excluded, but three SOD1 mutations were found. This is not surprising, since a genetic overlap between ALS and PMA has been previously found^45^.

### Prevalence and incidence

To the best of our knowledge, this is the first study in which incidence and prevalence of MND, including ALS, PMA and PLS, have been assessed.

In line with the literature, ALS was the most frequent MND, comprising 85.5–96.7% of the MND patients. The prevalence and incidence estimates of both MND and ALS is within the ranges described in the literature (a prevalence of between 4.1 and 8.4 per 100.000 person-year and an incidence of between 0.6 and 3.8 per 100.000 person-year^6^).

More specifically, the prevalence estimates in this study were very similar to those reported by Chiò et al.^9^ (median prevalence of 4.48; interquartile range, IQR, 3.03–6.70) and fairly similar to those found by Orsini et al.^10^ (a prevalence of between 4 and 6 per 100,000 inhabitants); Tesauro et al.^8^ (5 per 100,000 inhabitants); Pradas et al.^46^ (prevalence for Catalonia of 5.4 per 100,000 inhabitants); and Belbasis et al*.*^7^ (5.4 per 100,000 inhabitants).

The incidence estimates found in this study were almost identical to those reported by Marin et al*.*^18^ (standardised incidence of 1.68, 95% CI 1.50–1.85 all per 100.000 person-year) and in the range found by Chiò et al*.*^9^ (median incidence in Europe of 2.08 per 100,000 person-year, IQR: 1.47–2.43); Zarei et al.^18^ (incidence in Europe of between 1.5 and 2.7 per 100,000 person-year) and Santurtun et al.^47^ (incidence in Spain of between 1 and 3 cases per 100,000 person-year).

However, the incidence estimates found here differ from those described by Pradas et al.^46^, who estimated an incidence for Catalonia below the credibility interval found in this study (standardised incidence of 1.4 per 100,000 person-year vs. 1.682 per 100,000 person-year, respectively).

Notably, these comparisons must be made with caution. The estimators for prevalence and incidence present a huge heterogeneity. This could be due to the heterogeneity of the disease itself, to the structure of age and sex of the population studied, to geographical variables, to environmental factors, to the period under study (2011–2019 in this case, 1995–2011 in Chiò et al.^9^, the decade of the 1990s in Zarei et al.^19^ and 2013 in Santurtun et al.^47^ and Pradas et al.^46^), to the different diagnostic criteria, to different clinical practices, to the genetic predisposition of the subjects, to the type and sources of data used, to the type of design (prospective-retrospective, cohort-transversal study), and/or to the method used to calculate the prevalence and incidence (crude, standardised and multivariate models). An additional significant problem is that many of the authors do not explain the method they used to estimate the incidence and the prevalence.

Regarding PMA and PLS, there have been no previous epidemiological studies on these diagnostic categories, although their incidence has been estimated at < 0.1 per 100,000^2,43^. The estimated incidence found in this study ranges from 0.2 to 0.6 in both categories, suggesting a higher incidence than previously thought. However, these point estimates must be considered with caution given the wide confidence intervals.

In the present study, the point estimates of both prevalence and incidence for Valencia were, in all cases, lower than those for Catalonia. Given that the structure of age and sex was similar for both regions, and that economic deprivation was controlled for (possible risk factor), we believe that such differences are not due to the presence of additional risk factors because these are two Mediterranean regions with similar characteristics. These differences could be attributed to the different coverage provided by the referral hospitals in the two regions. While health care is public and universal in both regions, they each have their own organisation in terms of care, which could alter patient referral criteria and percentages. Furthermore, the population in Valencia is more dispersed geographically (39.6% of its inhabitants live in the metropolitan area of Valencia vs 71.5% living in the metropolitan area of Barcelona in Catalonia), thus (possibly) limiting access to the La Fe Hospital’s UFMN. Despite this, differences in prevalence and incidence between areas were not statistically significant, since there was a considerable overlap in the confidence intervals. This suggests that the methodological approach is robust enough to account for local differences.

This study has some limitations. First, despite clinical data being prospectively obtained, some demographic data were collected retrospectively. This retrospective feature may have implied that the point estimators that we have obtained have been lower than those obtained in prospective cohorts of other European studies. Second, some patients that did not meet the criteria for ALS, PLS or PMA (according to the definition used here) when the study was carried out. These patients, while having an MND, were not included in the present study since they did not yet have a confirmed diagnosis which would have enabled them to be classified into one of the categories. Therefore, both incidence and prevalence may be greater than those estimated. However, this limitation applies for all previous epidemiological studies regardless of the definition used. Moreover, for this study we used the most recent consensus definitions. Third, the studied period is different in the two hospitals and the coverage of cases may also be different. Nonetheless, the method is sufficiently robust to give similar results, at least regarding the interval estimate. Last, when the population at risk is very small, the point estimators can be relatively unstable. Therefore, it is better to produce aggregated results and to interpret Figs. 3 and 4 with caution. Furthermore, point estimates must never be interpreted in isolation but rather together with their interval estimators (as was done throughout this study) which, with a certain probability, contain the true value of the parameter (i.e. prevalence and incidence).

This paper has several strengths. First, this is the first study to estimate the prevalence and incidence of PLS and PMA. Second, contrary to the studies based on information systems, here the diagnosis of MND and its variants was supervised by specialists in MND in leading hospitals, thus minimizing the possibility of diagnostic errors. Third, in Spain there are only two studies estimating the prevalence and incidence of ALS using information from prior to 2011, neither of which provide estimates of MND according to the different diagnostic categories cited previously or are differentiated by region. Fourth, we propose an unbiased estimate of prevalence and incidence when, as in our case, there is selection bias due to the lack of random samples and, in addition, this selection bias is endogenous. Moreover, the sample used in this study comprised a large number of patients from two Spanish referral hospitals. Last, despite the different coverage in the two hospitals analysed, the fact that both the prevalence and the incidence estimators (by interval) overlapped can be taken as in indication that the method proposed enables the selection bias to be controlled for.

## Conclusion

In summary, within the context of RWD, we have used an original estimation method which has allowed us to control for the selection bias resulting from not using random samples of the population. With this method, confounders, both observed and unobserved, individually and contextually, were appropriately controlled. Using this method, estimators were obtained of both the prevalence and the incidence of MND and its different categories: prevalence of MND, with a 95% probability, between 3.990 and 6.334 per 100,000 inhabitants; between 3.248 and 5.120 per 100,000 inhabitants for ALS; between 0.065 and 0.634 per 100,000 inhabitants for PMA; and between 0.046 and 1.896 per 100,000 inhabitants for PLS; incidence of MND, between 1.682 and 2.165 per 100,000 person/years; between 1.351 and 1.754 per 100,000 person-years for ALS; between 0.225 and 0.628 per 100,000 person-years for PMA; and between 0.409 and 0.544 per 100,000 person-year for PLS.

Using an original estimation method based on RWD, the prevalence and incidence of ALS and, for the first time, of PMA and PLS, in two Spanish Mediterranean regions was obtained. The data produced are similar to those of other regions and do not differ greatly from data reported previously for ALS, suggesting that the proposed method is robust and that neither region presents differential risk or protective factors.

## Data Availability

Due to the ethical (in accordance with the protocol approved by the Clinical Research Ethics Committee (CEIC) of the University Hospital of Bellvitge) and legal (the provisions of the Spanish Law on Data Protection, Fundamental Law 15/1999 of 13 December on the Protection of Personal Data, article 7.3) restrictions on the transfer of data to third parties, the data (appropriately anonymized) is available to all interested researchers upon request to Maria A. Barceló (antonia.barcelo@udg.edu).

## Code availability

The code used is available upon request to the authors.
